# An increased risk of urinary tract infection precedes development of primary biliary cirrhosis

**DOI:** 10.1186/1471-230X-11-95

**Published:** 2011-08-26

**Authors:** Fumi K Varyani, Joe West, Timothy R Card

**Affiliations:** 1 Division of Epidemiology and Public Health, University of Nottingham, Clinical Sciences Building Phase 2, Hucknall Road, Nottingham, NG5 1PB, UK; 2 Nottingham Digestive Diseases Centre Biomedical Research Unit, University of Nottingham, Queens Medical Centre, Derby Road, Nottingham, NG7 2UH, UK; 3 Department of Gastroenterology, King's Mill Hospital, Mansfield Road, Sutton-In-Ashfield, NG17 4JL, UK

**Keywords:** PBC, UTI, Aetiology, AMA, Antimitochondrial

## Abstract

**Background:**

Primary Biliary Cirrhosis is known to be associated with Urinary Tract Infections (UTIs), but whether these precede or follow the liver disease is unclear. We have therefore attempted to determine whether UTIs are more common in people with Primary Biliary Cirrhosis (PBC) prior to their diagnosis.

**Methods:**

We conducted a case control study in the General Practice Research Database. All cases of PBC first recorded at least one year after entry to the dataset were selected along with up to 10 controls matched for age, sex. A second unmatched control group who had Chronic Liver Diseases but not PBC were chosen. The main exposures studied were the occurrence of Urinary tract infections and pyelonephritis at least one or at least five years before diagnosis. We also performed an analysis restricted to those younger than 55 at diagnosis, as we hypothesized the relationship to be stronger in the younger age group.

**Results:**

PBC is associated with UTI prior to diagnosis, OR 1.50 (CI 1.26-1.78), which was similar 5 years prior to diagnosis and after adjusting for smoking. The strongest relationships were observed in pyelonephritis exposures five years before diagnosis in cases under 55 years: adjusted odds ratios were 2.60 (1.02-6.63) in comparison with matched general population controls and adjusted odds ratios were OR 2.45 (1.02-5.59) in the comparison with chronic liver disease controls.

**Conclusions:**

We found that the association between urosepsis and PBC is specific to this disease and precedes the diagnosis of PBC in a manner not previously observed in human data. This is consistent with a causal relationship.

## Background

Primary Biliary Cirrhosis (PBC) is an idiopathic chronic liver disease associated with destruction of small intrahepatic bile ducts [1]. Both genetic [2] and environmental factors [3] have been implicated in its aetiology but no complete explanation is yet accepted. It seems however likely that the aetiology is a complex interplay between multiple factors.

There has been recent epidemiological interest in the spatio-temporal clustering of cases, which might indicate involvement of a transient toxic or transmissible agent [4,5]. The agent involved however is as yet uncertain. A number of potential infectious precipitants have however previously been considered [6] including organisms such as Mycoplasma [7] and *Novosphingobium.aromaticivorans*[8]. Another group of infections which have received attention are Urinary Tract Infections (UTIs)[9]. A number of studies have suggested a specific link with *Escherichia .Coli*[10,11] which is the commonest urinary isolate in the UK [12], and it has been suggested that this may occur via a mechanism of molecular mimicry [13]. Epidemiologically it has been known for some time, that patients with PBC are at increased risk of urinary tract infection after diagnosis [14] and four case control studies in recent years have confirmed the presence of an association [15-18]. They have been unable however to clearly suggest whether it is a cause or consequence of PBC since all of these studies have been retrospective case control studies with exposure to UTI ascertained by questionnaire at unspecified points in the disease course; i.e. at any time during life. In each study therefore, it is possible that the UTIs recorded occurred after diagnosis, rather than before onset of the disease, and each was inevitably susceptible to recall bias. Since an exposure can be the cause only of outcomes which occur after it, this means that one cannot ascribe causality based upon these studies. Further though Burroughs and colleagues, in their pioneering work in this area, showed that after diagnosis PBC was associated with UTI in a manner not seen for other chronic liver disease [14], such specificity would also need to be shown for any antecedent association if it were to be believed causal.

We have therefore set out to establish whether the epidemiology of the relationship between PBC and UTI is consistent with a causal relationship both by better defining the temporal relationship between PBC and UTI and by establishing whether any relationship is specific to PBC among liver disease. To achieve this we have carried out a large population based case-control study nested in the United Kingdom General Practice Research Database.

## Methods

The United Kingdom General Practice Research Database (GPRD) is a large longitudinal database of primary care records with data from 1987 until the present [19]. It has over 50 million patient years of data and is constantly being updated by primary care physicians. Participating practices contribute anonymised data for patients including demographics, all major illnesses, diagnoses, prescriptions, their indications and reason for withdrawal [19]. Data, which then meets the quality indicators set by GPRD is termed "up to standard" and it is this data which has been used to derive our cases, controls, exposures and covariates. It has been validated by several studies as a high quality dataset which accurately reflects a range of diagnoses [19,20].

### Study design

We constructed an individually matched case control study nested within GPRD, and using the same cases a second unmatched case control study. Cases were all incident cases of PBC in GPRD. These were defined as those with READ/OXMIS codes for PBC (READ Primary Biliary Cirrhosis or OXMIS code PRIMARY BILIARY CIRRHOSIS (LIVER)) or Anti-mitochondrial antibody (AMA) alongside another code for chronic liver disease or cholestatic liver function tests with AMA or PBC first recorded at least one year after the start of the subject's up to standard data from January 1987 to October 2008. General population controls matched for age, sex and practice were selected at a ratio of approximately 10:1. A second group of unmatched controls who had Chronic Liver Diseases (CLD) but not PBC were selected to permit assessment as to whether findings were specific to PBC or were characteristic of all chronic liver diseases.

### Exposures

The primary exposure of interest was the occurrence of urinary tract infection prior to diagnosis of PBC. To study this, we abstracted data on the occurrence of episodes of Urinary Tract Infections (UTI) and of Pyelonephritis from the GPRD record using READ and OXMIS codes for diagnoses. These events were used to categorise subjects as having ever had a UTI or Pyelonephritis occurring at least a year before diagnosis, and occurring at least five years before diagnosis.

### Potential confounders

We considered age, gender, smoking status, and diabetes as potential confounders. For the general population control group, the data was matched for age and gender which are both known to be important risk factors in PBC [21]. For the analysis using the chronic liver control disease group we divided age into 4 bands which produced rough quartiles (less than 45 years at diagnosis, 45-55 years, 55-60 years and over 60 years). Smoking, which is known to be more prevalent in patients with PBC and is considered to be an important risk factor for it [15,16], was categorised as ever smoked, never smoked or missing data on smoking at least a year before diagnosis. Finally diabetes as a known risk factor for the development of urinary tract infections [22], was assessed as a potential confounder categorized as ever or never having a diagnosis of diabetes.

### Analysis

All analyses were conducted using STATA10.1/SE. We conducted initial descriptive analyses of the data to produce summary measures of the primary and potentially confounding exposures. Univariate analyses using both general population and CLD controls were conducted using conditional logistic regression for the matched control group and logistic regression for the unmatched controls. We then went on to conduct multivariate regression analyses to assess the effect of smoking in the matched controls. As the CLD controls were unmatched, we also adjusted for age and gender to remove confounding by them. Age and gender were retained in the final model on an a priori basis, but other confounders only if their addition or removal altered the odds ratio by at least 10%. Finally a further stratified analysis was performed by dividing patients into those older than or aged 55 years at diagnosis, and those who were younger than 55 at diagnosis to examine any possible interaction with age. We hypothesised that in post menopausal women, the likelihood that a recorded UTI would accurately reflect infection with *Escherichia .Coli *was lower due to the potential for misdiagnosis of dysuria due to reduced oestrogen levels in this group. Hence we expected to find a weaker relationship in those over 55.

### Role of the funding source

The data used to conduct this study was provided via an MRC license. Dr Varyani is an Academic Clinical Fellow funded by the UK NIHR, Dr Card is funded via a HEFCE Senior Lectureship Award and Dr West is an NIHR clinician scientist. None of these funders had any role in the design, conduct, interpretation or writing up of this study.

### Ethics

The GPRD Group has ethical approval from a Multi-centre Research Ethics Committee covering all purely observational research using GPRD data (including the current study). The protocol for this project was approved by the Independent Scientific Advisory Committee of the GPRD (protocol number 08_068).

## Results

We had in total 800 subjects with PBC, 7991 general population matched controls and 12137 unmatched chronic liver disease controls. Overall 87% of PBC cases were female and mean age at diagnosis was 63 years. Cases had a mean follow up time of 6.5 years and both sets of controls had a similar amount of follow up time. The Chronic Liver Disease cohort was younger with a mean age of 51 years and 40% were female. 44% of the PBC patients had ever smoked compared to 37% of the general population controls and 56% of the chronic liver disease control group. 12% of the PBC patients had ever had a diagnosis of diabetes compared with 9% of the controls and 21% of the CLD controls (Table 1). We found smoking to be associated with prior UTI exposure and with PBC. Diabetes however was not significantly associated with UTI or pyelonephritis and its inclusion in multivariate models did not appreciably alter the effect estimates for UTI or pyelonephritis. It was hence not included in the final models.

**Table 1:** Baseline Characteristics for population in study

	Cases	Match	Chronic Liver Disease
N	800	7,991	12,137

% Female	87	87	40

Mean age	63	63	51

% > 55 years	72.3	72.7	49.6

Smoker			

Never	46.3	49.7	34.1

Ever	43.8	36.5	55.5

Missing	10.0	13.8	10.4

% Diabetes	12.3	8.9	20.5

Mean followup	6.53	6.25	7.00

Cases are PBC and Match are general population controls matched for age and sex. Chronic liver disease are those with chronic liver disease who do not have a diagnosis of PBC, these are unmatched controls

Overall 229 (29%) of PBC patients had a UTI recorded in the dataset at least one year before diagnosis, compared to 1737 (22%) of the general population controls (Table 2). The odds of having a prior diagnosis of UTI at least one year before diagnosis were 1.50 (CI 1.26-1.78) times as high for PBC cases as for matched controls. Results were similar for UTIs at least five years before diagnosis and when the analysis was adjusted for the potential confounding effects of smoking. When these analyses were repeated restricting the exposure to diagnoses of pyelonephritis instead of all UTIs, we found 14 (1.75%) of the PBC patients had a prior episode of pyelonephritis compared to 79 (0.99%) of general population matched controls. This corresponded to an unadjusted OR of 1.79 (CI 1.01-3.19) which was similar when adjusted for smoking. Odds ratios for Pyelonephritis five years prior to diagnosis were 1.55 (CI 0.79-3.05), however numbers of cases were small with only 10 (1.25%) of cases and 66 (0.83%) of controls having had Pyelonephritis five years prior to diagnosis. Using the controls with chronic liver disease produced on univariate analysis slightly larger estimates of effect which were reduced when corrected for confounding by age and gender in UTIs at least a year before diagnosis. However, these estimates were maintained in UTIs five years before diagnosis even after adjustment, and were slightly stronger OR 2.26 (CI 1.28-3.98). Restricting the chronic liver disease comparison to pyelonephritis exposures instead of all UTIs found 85 (0.70%) of controls with prior pyelonephritis. The unadjusted OR was 2.15 (CI 1.06-3.23) but this changed to 2.35 (CI 0.54-10.24) after adjustment, and when restricting the comparison to pyelonephritis exposures five years prior to diagnosis, only 85 (0.70%) of the controls had an exposure, giving an unadjusted OR of 1.79 (CI 0.93-3.47) but an adjusted OR of 5.77(CI 1.15-29.08).

**Table 2:** Odds of UTIs or of Pyelonephritis occurring at least one or at least five years before diagnosis

		**Matched controls**.			Chronic liver disease controls		
**Exposure**	**Case (%)**	**Control (%)**	**OR (CI)**	**Adjusted OR (CI)**	**Control (%)**	**OR (CI)**	**Adjusted OR (CI)**

UTI > 1 year	229 (28.63)	1737 (21.74)	1.50 (1.26-1.78)	1.49 (1.25-1.77)	2044 (16.84)	1.98 (1.69-2.33)	1.53 (0.92-2.55)

UTI > 5 years	150 (18.75)	1,095 (13.70)	1.52 (1.24-1.85)	1.50 (1.23-1.84)	1281 (10.55)	1.96 (1.62-2.36)	2.26 (1.28-3.98)

Pyelonephritis > 1 year	14 (1.75)	79 (0.99)	1.79 (1.01-3.19)	1.83 (1.03-3.26)	116 (0.96)	2.15 (1.06-3.23)	2.35 (0.54-10.24)

Pyelonephritis > 5 years	10 (1.25)	66 (0.83)	1.55 (0.79-3.05)	1.55 (0.79-3.05)	85 (0.70)	1.79 (0.93-3.47)	5.77 (1.15-29.08)

*The adjusted models are adjusted for diabetes in the comparison to matched controls, and age gender and diabetes in the comparison to chronic liver disease controls*.

Stratification of UTI occurrence by age groups shows some interaction between age, frequency of UTIs and PBC. Formally testing this in our multivariate model demonstrated this interaction to be significant in UTIs five years prior to PBC diagnosis (p < 0.05), although a trend was observable in the other exposures assessed. We therefore conducted further analyses stratified by age. Of those patients diagnosed with PBC before the age of 55, 68 (31%) had a prior diagnosis of UTI (at least one year prior to diagnosis), compared to 433 (20%) of age and sex matched controls. This corresponds to PBC patients in this age group having odds of prior diagnosis of UTI 1.98 fold higher than controls (CI 1.43-2.75). This figure was virtually unchanged by adjustment of any confounding by smoking, but fell to an OR of 1.36 (1.11-1.66) among those aged 55 or over at diagnosis. When comparing to chronic liver disease controls there was clear confounding by age and gender, and after adjustment for this, the relationships were weaker and ceased to be significant (Table 3).

**Table 3:** Odds of UTIs and odds of pyelonephritis occurring at least one or at least five years before diagnosis stratified by age

		**Matched controls**.			Chronic liver disease controls		
**Age groups**	**Case (%)**	**Control (%)**	**OR (CI)**	**Adjusted OR (CI)**	**Control (%)**	**OR (CI)**	**Adjusted OR (CI)**

UTIs at least one year before diagnosis							

< 55	68 (31.2)	433 (19.9)	1.98 (1.43-2.75)	1.97 (1.42-2.74)	865 (14.1)	2.76 (2.06-3.71)	1.24 (0.91-1.68)

> = 55	161 (27.7)	1304 (22.4)	1.36 (1.11-1.66)	1.34 (1.10-1.64)	1179 (19.6)	1.56 (1.29-1.90)	0.90 (0.74-1.10)

UTIs at least five years before diagnosis							

< 55	53 (24.3)	280 (12.9)	2.39 (1.67-3.42)	2.39 (1.67-3.42)	526 (8.3)	3.43 (2.48-4.73)	1.61 (1.15-2.25)

> = 55	97 (16.7)	815 (14.02)	1.25 (0.98-1.60)	1.24 (0.97-1.58)	755 (12.6)	1.39 (1.10-1.75)	0.83 (0.65-1.05)

Pyelonephritis at least one year before diagnosis							

< 55	7 (3.21)	34 (1.56)	2.13 (0.92-4.91)	2.16 (0.93-5.02)	60 (0.98)	3.36 (1.52-7.44)	1.87 (0.83-4.19)

> = 55	7 (1.20)	45 (0.77)	1.56 (0.70-3.46)	1.59 (0.71-3.55)	56 (0.93)	1.29 (0.59-2.85)	0.82 (0.37-1.83)

Pyelonephritis at least five years before diagnosis							

< 55	6 (2.75)	25 (1.15)	2.54 (1.00-6.46)	2.60 (1.02-6.63)	39 (0.64)	4.42 (1.85-10.56)	2.45 (1.02-5.95)

> = 55	4 (0.69)	41 (0.71)	0.98 (0.35-2.72)	0.99 (0.35-2.76)	46 (0.77)	0.90 (0.32-2.50)	0.58 (0.20-1.62)

*The adjusted models are adjusted for smoking in the comparison to matched controls, and with gender and smoking in the comparison to chronic liver disease controls*.

For comparison with both sets of controls, these relationships were similar if only UTIs at least 5 years before diagnosis were considered (adjusted OR in those under 55 of 2.39 (1.67-3.42) for matched and 1.61 (1.15-2.25) for chronic liver disease controls). Adjusted ORs for the relationship with Pyelonephritis in those under 55 were 2.60 (1.02-6.63) for matched and 2.45 (1.02-5.59) for chronic liver disease controls (Table 3).

## Discussion

Our study has shown clearly that UTI precedes the diagnosis of PBC and that this relationship is particularly strong in younger people with PBC. Overall, people with PBC were 50% more likely to have had a UTI previously than their matched controls; this relationship was maintained five years prior to diagnosis. In addition, people with PBC are at greater risk of having a prior UTI than people with chronic liver disease five years prior to diagnosis.

Though an association between PBC and UTI has previously been demonstrated, this study moves our knowledge forward in three important respects. Firstly, though due to the likely long preclinical course of PBC in many cases, we cannot be sure that UTI predates the onset of PBC, our data is the first to show evidence of an association prior to diagnosis that extends back at least to 5 years. This is of enormous importance since, only if UTI does precede PBC can this association be causative. Secondly we have shown that not all diagnoses of UTI have equal association with the risk of PBC. UTIs diagnosed in younger age groups, may be associated with greater risk. Thirdly, for UTIs and Pyelonephritis five years prior to diagnosis, we have shown that this association is unlikely to be due to better ascertainment of UTIs or chronic ill health among people with PBC as such a mechanism should apply equally to other liver diseases.

The current study is, in many ways far stronger than those which preceded it in other ways as well. Being population based, its results will be generalisable to patients cared for in UK general practice (the population from which it is drawn), and therefore in essence to the population of the UK. As it is based upon prospectively recorded data, there is no potential for recall bias and since all potential cases for the study were selected, and controls were chosen in an unbiased manner, selection bias also cannot realistically occur.

The great weakness of this as of any study based on an anonymised clinical records database is the potential for error in the definition of both outcomes and exposures which cannot be validated. For the current outcome of PBC, we do not have great concern as to error as we have previously shown that the epidemiology of PBC when described in this database closely mirrors the best modern conventional epidemiological studies available in the UK population [21,23-26]. The possibility of error in the coding of UTI needs however far closer attention. One should first note that UTI is itself in this study in effect being used as a proxy for *E. Coli *infection since this is the commonest UK isolate accounting for about 80% of cases [12] (our hypothesis is of the induction of autoimmunity by molecular mimicry by that organism). In addition, a clinical diagnosis (which for most of the recorded exposures is not supported by laboratory evidence) of UTI is a proxy for a true UTI. We have attempted to address this uncertainty in two manners. Firstly we have looked at pyelonephritis as a subset of UTIs far less likely to have been diagnosed without good evidence of urinary tract infection. This produced despite limited power and therefore a lack of significance generally greater estimates of effect than found for uncomplicated UTI. Secondly we considered that some urinary symptoms diagnosed as UTI in the post menopausal might in reality merely represent oestrogen deficiency of the urothelial membranes since this has been shown to be reversibly related to symptoms of urinary frequency and stress incontinence [27]. We therefore stratified by age hypothesising that the diagnosis would be more secure in the young. Again we found greater effect where the diagnosis was likely to be more secure. Taken together we believe these results suggest that if anything the error in coding of UTI is likely to have caused a null bias (tending to obscure a true association), which will have been less marked in those with pyelonephritis or UTIs at earlier age.

Our study has, of course, other weaknesses than that previously alluded to however. The greatest of these is that we have data only for a fairly limited time period, and cannot therefore look more than 5-10 years back from the diagnosis of PBC. In addition, we have no way to determine the time at which AMA positivity, or derangement of liver function first occurred, and so though this study is perhaps the best evidence yet that UTIs predispose to PBC rather than vice versa, it cannot on its own be regarded as conclusive. In support of this suggestion there is however some laboratory, experimental and human evidence regarding the development of AMAs and PBC in relation to infections. Though the development of AMAs is not exclusive to E Coli infection (it occurs occurs also with xenobiotics and other organisms [7,8,28]), a number of strands of evidence show its potential importance in this context. Examples of this include rabbits immunised with rough mutants of E.Coli which developed antibodies to AMA reactive components [29], mice infected with recurrent UTIs which developed AMAs and histology similar to early PBC [30] and the greater proportion of people with recurrent UTIs found to be AMA positive compared to the general population [31].

## Conclusion

In conclusion, we have demonstrated a strong association between UTI and subsequent diagnosis of PBC, that this association goes back at least five years before diagnosis and that it is stronger in younger subjects. In addition, this finding was specific to PBC when compared to other chronic liver diseases. This work provides the strongest evidence yet produced that this relationship is indeed a causal one.

## Abbreviations

AMA: Anti-mitochondrial antibody; CI: 95% Confidence interval; CLD: Chronic Liver Diseases; GPRD: General Practice Research Database; OR: Odds Ratio; PBC: Primary Biliary Cirrhosis; PDC-E2: Pyruvate dehydrogenase complex E2 subunit; UTI: Urinary Tract Infection.

## Competing interests

The authors declare that they have no competing interests.

## Authors' contributions

FKV under supervision by TRC and JW wrote the application for the data, managed the data, undertook the analysis, wrote the first draft, and contributed to the third and all subsequent drafts. JW contributed to supervision of FKV, data management, analysis and interpretation, and contributed to the writing of the third and all subsequent drafts. TRC conceived the project, contributed to supervision of FKV, data management, analysis and interpretation, wrote the second draft and contributed to the writing of the third and all subsequent drafts.

All authors read and approved the manuscript.

## Pre-publication history

The pre-publication history for this paper can be accessed here:

http://www.biomedcentral.com/1471-230X/11/95/prepub
